# Impact of Time-To-Treatment on Outcomes in Autoimmune Membranous Nephropathy

**DOI:** 10.1016/j.ekir.2025.04.005

**Published:** 2025-04-09

**Authors:** Patrick Hamilton, Sebastian Bate, Omar Ragy, Mrityunjay Hiremath, Samar Bukhari, Anirudh Rao, Arif Khwaja, Durga Anil K Kanigicherla

**Affiliations:** 1 Manchester Institute of Nephrology and Transplantation, Manchester University NHS Foundation Trust, Manchester, UK; 2 Wellcome Center for Cell-Matrix Research, Division of Cell Matrix Biology and Regenerative Medicine, School of Biological Sciences, Faculty of Biology Medicine and Health, University of Manchester, Manchester, UK; 3 Research and Innovation, Manchester Academic Health Science Center, Manchester University NHS Foundation Trust, Manchester, UK; 4 Center for Biostatistics, University of Manchester, Manchester, UK; 5 Renal Department, Liverpool University Hospitals NHS Foundation Trust, Liverpool, UK; 6 Renal Department, Sheffield Teaching Hospitals NHS Foundation Trust, Sheffield, UK

**Keywords:** anti-PLA2R, membranous nephropathy, nephrotic syndrome

## Abstract

**Introduction:**

Despite new treatments, kidney dysfunction in autoimmune membranous nephropathy (aMN) remains challenging. Immunosuppression is initiated after "watchful-wait" to avoid side effects in patients who might achieve spontaneous remission; however, the impact of this approach on kidney function is unclear.

**Methods:**

This was a retrospective longitudinal cohort study of patients with new consecutive aMN from 2003 to 2019 from 3 UK centers. Patients were assigned to 5 categories, *a priori* based on baseline urinary protein-to-creatinine ratio (uPCR) and estimated glomerular filtration rate (eGFR). The analysis investigated change in eGFR based on these risk categories. Primary outcomes were spontaneous partial remission (SPR), rate of eGFR change before immunosuppression, and chronic kidney disease (CKD) stage 5 (CKD5). The secondary outcome was progression (composite of doubling serum creatinine, CKD5, and death). Cox proportional hazard models were used to assess association of outcomes with baseline categories.

**Results:**

A total of 312 patients were included with 5.0 years median follow-up from diagnosis. A significant association was found between waiting time to immunosuppression and decline in eGFR (*P* < 0.001) in patients who received immunosuppression. When change was regressed against time, eGFR loss was equivalent to 7.7 ml/min per 1.73 m^2^/yr of watchful wait. In the low-risk group, 71% achieved SPR and 2.4% progressed to CKD5, whereas in the high-2 risk group, 20% achieved SPR, and 25% developed CKD5. The strongest predictor of progression to CKD5 was the eGFR at the start of immunosuppression treatment, regardless of baseline function.

**Conclusion:**

In patients with aMN requiring immunosuppression, delayed treatment leads to worse kidney outcomes. A simple categorization using baseline eGFR and uPCR can help predict spontaneous remission and potential kidney function decline.

## Graphical abstract

**Figure:**
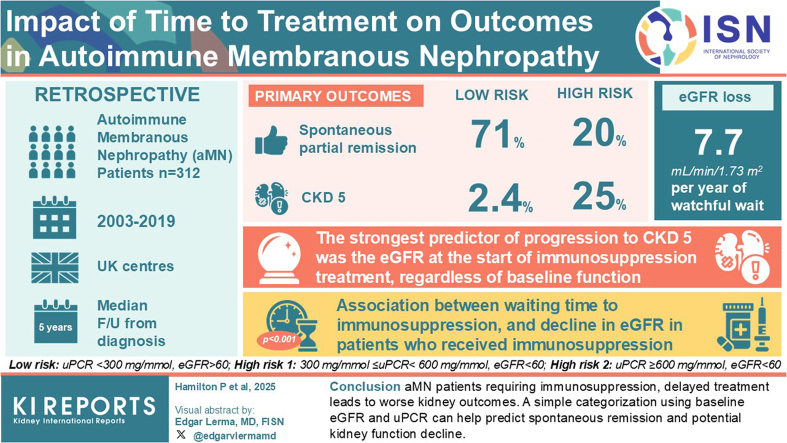

aMN is an autoimmune condition with antibodies to podocyte antigens detected in a majority of patients.^1^ The clinical spectrum of aMN is broad, with patients presenting with minimal symptoms through to significant nephrotic syndrome. Longitudinal studies have shown that approximately one-third of patients enter spontaneous remission^2^^,^^3^; however, the disease can progress, leading to end-stage kidney disease in about 20% to 40% over a 10- to 15-year period.^4^, ^5^, ^6^ Advancements in this understanding of aMN's pathophysiology have helped in supporting diagnosis and management. However, translation into directed therapy has yet to be fully realized and the characteristics of disease progression are still not well-understood. The difficulty in predicting disease progression, in combination with the side-effect burden of the most widely used treatment regimens, has led guidelines to suggest a period of monitoring in many patients before embarking on immunosuppression.^7^^,^^8^ This watchful-wait approach was borne out of necessity in the era of limited understanding of biomarkers of disease activity and limited treatment options with higher adverse effect profile. Features of kidney parameters during such an observation and whether this watchful waiting in itself could be deleterious has not been well-studied.

Although prognostication remains an imprecise science there is a recognition that those with a rapid decline in kidney function are at higher risk of progression of kidney disease. The guidelines suggest an increased frequency of monitoring every few months for loss of kidney function^8^; however, such decline only becomes evident in retrospect. This would be of immense relevance if the loss of kidney function were to be irreversible. With less toxic therapies such as rituximab increasingly available, using a risk stratification for patients based on characteristics at presentation could alter the risk-benefit profile for treatment and could potentially allow patients to avoid long term morbidity with earlier treatment.

In this study, we used data from 3 tertiary referral centers in the UK to study the rate of kidney function progression during the watchful-wait period, and assessed potential predictors of spontaneous remission, and kidney progression after the initiation of immunosuppression. The study aimed to highlight the implications of “watchful wait” and to identify tools that could help target a more pragmatic and personalized approach for when to initiate immunosuppressive treatment in aMN.

## Methods

This retrospective longitudinal cohort study included patients from 3 large specialist kidney centers in the north of England (Sheffield, Liverpool, and Manchester) and is the second study using data from the 3 sites. The majority of the methods are described by Ragy *et al.*^9^ In summary, the previous study, which was conducted in these 3 centers, included 222 patients presenting with nephrotic syndrome between January 2003 and July 2019. Patients were included if they had histologically confirmed MN or a positive serum anti–phospholipase A2 receptor (anti-PLA2R) antibody test (EUROIMMUN enzyme-linked immunosorbent assay > 20 RU/ml or immunofluorescence). Those with subnephrotic proteinuria or eGFR < 60 ml/min per 1.73 m^2^ were excluded. Baseline data included demographic details, eGFR, uPCR, and anti-PLA2R antibody status and titers. In that study, the primary outcome was time to progression (defined as doubling of serum creatinine, CKD5, or death), whereas the secondary outcomes included partial remission and time to immunosuppression initiation. Patients were analyzed in groups based on antibody-positive versus antibody-negative status and high-titer versus low-titer categories. Survival analyses were performed using Kaplan–Meier plots and Cox proportional hazards models, adjusted for eGFR and uPCR. Time-0 was defined as the date of biopsy or anti-PLA2R testing.

The cohort comprised consecutive patients presenting with new incident nephrotic syndrome from aMN between January 2003 and July 2019 and follow-up data recorded through to September 2021. Patients were included if their diagnosis of aMN was confirmed histologically and/or clinical diagnosis with positive anti-PLA2R. In patients without a biopsy, diagnosis was confirmed with an anti-PLA2R titer > 14 using the EUROIMMUN enzyme-linked immunosorbent assay, and no history of diabetes mellitus. Baseline diagnosis of aMN was made by nephrologists after appropriate history, examination, and autoimmune serology, and malignancy and systemic diseases were ruled out. We have excluded patients with subnephrotic proteinuria and an eGFR < 60 ml/min per 1.73 m^2^ to avoid other possible overriding pathologies alongside MN, which may confound the progression of kidney impairment. The study size was pragmatic and based on all eligible patients.

Baseline characteristics included demographic details at diagnosis, markers of disease with eGFR, uPCR at presentation, and anti-PLA2R test result (positive vs. negative and titers; the definition of positivity is available in Ragy *et al*^9^). Baseline values were defined as the laboratory values closest to the date of diagnosis. Before 2012, patients recruited in MN studies in Manchester (Ethics 06/Q1401/5 and 10/H1008/10) had their stored samples analyzed in retrospect for anti-PLA2R by enzyme-linked immunosorbent assay.

For the analysis of change in eGFR, those starting immunosuppression within 2 weeks were excluded from this analysis because their data would be considered too unstable to draw reliable conclusions. The coprimary end points were partial remission and CKD5 after immunosuppression. Partial remission was defined as uPCR < 350 mg/mmol and reduction of >50% compared with baseline. Spontaneous remission was defined as partial remission without immunosuppression. Progression was defined as a composite outcome of doubling of serum creatinine, CKD5, or death (whichever was earliest). Patients were able to have multiple outcomes, for example, partial remission followed by a relapse then progression, although some events were mutually exclusive, such as SPR and immunosuppression use. To avoid the bias of older age on death, we repeated our analysis focusing on kidney end points.

Based on the Kidney Disease Improving Global Outcomes guidelines, we assigned patients to *a priori* defined risk groups as follows:

• Low uPCR < 300 mg/mmol eGFR > 60 ml/min per 1.73 m^2^
• Moderate-1 300 mg/mmol ≤ uPCR < 600 mg/mmol eGFR ≥ 60 ml/min per 1.73 m^2^
• Moderate-2 uPCR ≥ 600 mg/mmol eGFR ≥ 60 ml/min per 1.73 m^2^
• High-1 300 mg/mmol ≤ uPCR < 600 mg/mmol eGFR < 60 ml/min per 1.73 m^2^
• High-2 uPCR ≥ 600 mg/mmol eGFR < 60 ml/min per 1.73 m^2^

The risk groups are based on the traditional cut-off of 300 mg/mmol for nephrotic syndrome^8^ and then a further split at 600 mg/mmol to denote severe proteinuria. These groups are easy to use and only depend on information that is available at diagnosis.

We applied survival methodology for data analyses. “time-0” or baseline was the date of the biopsy or anti-PLA2R test (if kidney biopsy was not performed), or for postimmunosuppression analyses, time of first immunosuppression. Patients were censored at death or last-follow-up. We used Cox proportional hazards testing to evaluate the association of the variable in question with outcomes. Harrell’s C and analysis of variance was used to assess model performance in Cox PH multivariable models .^10^^,^^11^ Unadjusted linear regression was used to assess the decline of eGFR over time from diagnosis to start of immunosuppression. Complete-case analysis was used; however, there was very little missing data. Subgroups by partition were not analyzed. There were no sensitivity analyses. We censored the use of immunosuppression in 1 model to elucidate the natural course of the disease. Survival analysis was presented using Kaplan–Meier plots. Survival analysis events were taken as time to spontaneous remission and time to the start of immunosuppression. Model performance was compared using concordance statistic and analysis of variance.^10^^,^^11^ Analyses for time to immunosuppression was done excluding any patients with previous immunosuppression use. Immunosuppression included glucocorticoids, calcineurin inhibitors, alkylating agents, mycophenolate mofetil, and rituximab, recorded as yes or no. Analyses excluded those with eGFR < 15 ml/min per 1.73 m^2^ at time-0.

Analysis was performed using R v4.2.1 (R Core Team, Vienna, Austria). *P* < 0.05 was considered significant. The study is reported in line with STROBE guidelines.^12^^,^^13^

## Results

A total of 312 consecutive patients were included in the study across the 3 centers. Among the total cohort, 10 patients (3%) were labeled as having a diagnosis of aMN without a kidney biopsy. These 10 patients all had positive EUROIMMUN enzyme-linked immunosorbent assay anti-PLA2R, eGFR > 60 ml/min per 1.73 m^2^ at diagnosis, and had no other features to point to an alternative diagnosis for nephrotic syndrome other than aMN. One hundred sixty-eight patients (53.8%) had proteinuria > 600 mg/mmol and included 93 patients (29.8%) with baseline eGFR ≥ 60 (moderate-2 risk group) and 75 patients (24%) with eGFR < 60 ml/min per 1.73 m^2^ (high-2 risk group).

**Figure 1:**
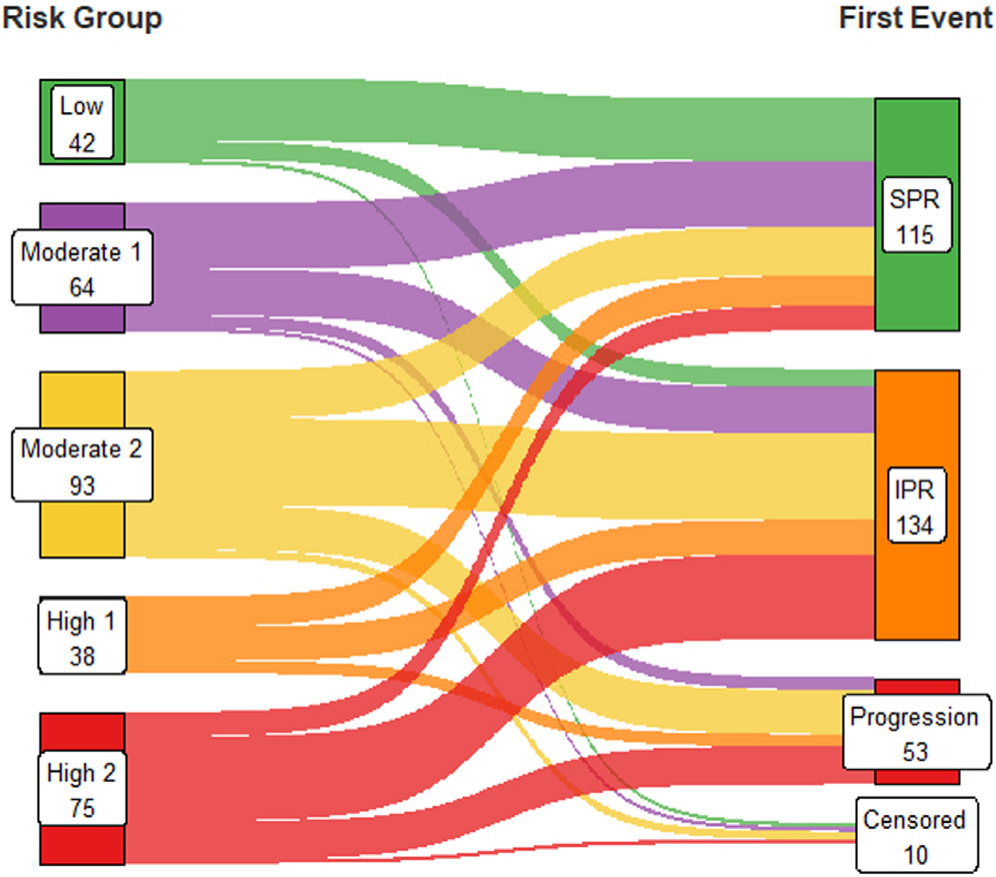
Sankey plot showing clinical outcomes based on risk group.

Baseline characteristics are presented in Table 1. Median eGFR was 69.6 ml/min per 1.73 m^2^ and uPCR was 631.8 mg/mmol at diagnosis. Of note, patients in high-1 and High-2 were older and there was a higher proportion of males in the high-2 group. Patients who achieved SPR had a higher eGFR at diagnosis than the groups that ended up requiring immunosuppression and the progression groups, which were similar. Initiation of first immunosuppression was at median eGFR of 49 ml/min per 1.73 m^2^ and uPCR at 675 mg/mmol (Table 2). Partial remission was observed in 258 (82.7%), with this being spontaneous in 118 patients (37.8%). In Figure 1, we show the first event in different risk groups, with most of the progression contributed by moderate-1, high-1, and high-2 risk groups. The likelihood of achieving rate of progression (composite outcome) was 26% in the whole cohort, with rates increasing from low risk to high risk (high-1 and high-2) groups (Table 3). Those without any event had shorter follow-up times and were more recent additions to the cohort.

**Table 1:** Demographics and biochemistry at baseline

Characteristics	All patients *N* = 312	Low *n* = 42	Moderate-1 *n* = 64	Moderate-2 *n* = 93	High-1 *n* = 38	High-2 *n* = 75
Age at diagnosis (yrs)						
Median (IQR)	61.9 (52.08–70.60)	60.2 (49.55–68.88)	58.0 (50.55–67.23)	55.4 (46.90–64.00)	70.0 (64.28–75.38)	67.5 (60.70–76.40)
Gender						
Male	216 (69.2%)	28 (66.7%)	41 (64.1%)	64 (68.8%)	21 (55.3%)	62 (82.7%)
Ethnicity						
White	254 (81.4%)	35 (83.3%)	50 (78.1%)	71 (76.3%)	33 (86.8%)	65 (86.7%)
Follow-up (yrs)						
Median (IQR)	5.0 (2.5–8.3)	5.9 (3.2–9.1)	5.2 (3.2–8.9)	6.6 (3.3–9.6)	4.6 (2.2–7.6)	3.4 (1.9–6.6)
Anti-PLA2R						
Positive	151 (48.4%)	21 (50.0%)	33 (51.6%)	43 (46.2%)	16 (42.1%)	38 (50.7%)
Negative	72 (23.1%)	11 (26.2%)	11 (17.2%)	20 (21.5%)	9 (23.7%)	21 (28.0%)
Not Done	89 (28.5%)	10 (23.8%)	20 (31.2%)	30 (32.3%)	13 (34.2%)	16 (21.3%)
eGFR, (ml/min per 1.73 m^2^)						
Median (IQR)	69.6 (46.00–88.00)	87.0 (73.33–94.75)	85.0 (70.43–92.25)	76.2 (70.00–94.00)	39.3 (29.21–49.04)	39.0 (32.00–48.00)
uPCR, (mg/mmol)						
Median (IQR)	631.8 (439.60–882.18)	248.8 (200.90–274.00)	448.0 (395.22–527.58)	823.2 (718.20–1036.00)	490.5 (445.38–541.75)	907.9 (743.05–1048.60)
Albumin (g/l)						
Median (IQR)	23.0 (18.00–28.00)	29.0 (26.25–33.00)	24.0 (21.00–27.00)	21.0 (17.00–26.00)	24.5 (21.00–27.75)	21.0 (17.00–26.00)

anti-PLA2R, anti–phospholipase A2 receptor; CKD5: chronic kidney disease stage 5; eGFR, estimated glomerular filtration rate; IQR, interquartile range; uPCR, urinary protein-to-creatinine ratio.

Low: uPCR < 350 mg/mmol and eGFR > 60 ml/min per 1.73 m^2^; Moderate-1: 350 mg/mmol ≤ uPCR < 600 mg/mmol and eGFR ≥ 60 ml/min per 1.73 m^2^; Moderate-2: uPCR ≥ 600 mg/mmol and eGFR ≥ 60 ml/min per 1.73 m^2^; High-1: 300 mg/mmol ≤ uPCR < 600 mg/mmol and eGFR < 60 ml/min per 1.73 m^2^; High-2: uPCR ≥ 600 mg/mmol and eGFR < 60 ml/min per 1.73 m^2^.

**Table 2:** eGFR and uPCR at initiation of immunosuppression

Parameters	All Patients	Low	Moderate-1	Moderate-2	High-1	High-2
	*N* = 186	*n* = 11	*n* = 32	*n* = 62	*n* = 23	*n* = 58
eGFR (ml/min per 1.73 m^2^)	49.0 (32.07–71.00)	78.5 (65.00–87.75)	65.0 (53.50–82.00)	66.5 (50.38–92.25)	30.0 (20.00–38.00)	35.3 (25.25–43.96)
uPCR (mg/mmol)	675.2 (445.20–915.60)	254.0 (207.20–405.00)	487.0 (348.38–557.85)	777.9 (622.47–971.25)	476.0 (382.50–753.55)	775.0 (588.35–966.00)
Albumin (g/l)	23.0 (18.00–28.00)	32.0 (29.25–34.75)	24.0 (19.50–28.00)	21.0 (18.00–26.00)	24.0 (18.50–28.00)	22.0 (17.25–27.75)
Time to IS (years)	0.3 (0.1–0.6)	0.4 (0.3–0.8)	0.5 (0.1–1.4)	0.3 (0.1–0.5)	0.2 (0.1–0.3)	0.3 (0.2–0.6)

eGFR, estimated glomerular filtration rate; IQR, interquartile range; uPCR, urinary protein-to-creatinine ratio.

Low: uPCR < 350 mg/mmol and eGFR > 60 ml/min per 1.73 m^2^; Moderate-1: 350 mg/mmol ≤ uPCR < 600 mg/mmol and eGFR ≥ 60 ml/min per 1.73 m^2^; Moderate-2: uPCR ≥ 600 mg/mmol and eGFR ≥ 60 ml/min per 1.73 m^2^; High-1: 300 mg/mmol ≤ uPCR < 600 mg/mmol and eGFR < 60 ml/min per 1.73 m^2^; High-2: uPCR ≥ 600 mg/mmol and eGFR < 60 ml/min per 1.73 m^2^.

All values presented as median (IQR).

**Table 3:** Clinical outcomes stratified by risk group

Clinical Outcomes	All Patients *N* = 312	Low *n* = 42	Moderate 1 *n* = 64	Moderate 2 *n* = 93	High 1 *n* = 38	High 2 *n* = 75
PR	258 (82.7%)	38 (90.5%)	55 (85.9%)	72 (77.4%)	31 (81.6%)	62 (82.7%)
SPR	118 (37.8%)	30 (71.4%)	31 (48.4%)	28 (30.1%)	14 (36.8%)	15 (20.0%)
IS	186 (59.6%)	11 (26.2%)	32 (50.0%)	62 (66.7%)	23 (60.5%)	58 (77.3%)
IPR	140/186 (75.3%)	8/11 (72.7%)	24/32 (75.0%)	44/62 (71.0%)	17/23 (73.9%)	47/58 (81.0%)
Progression	81 (26.0%)	4 (9.5%)	8 (12.5%)	26 (28.0%)	16 (42.1%)	27 (36.0%)
DSC	78 (25.0%)	4 (9.5%)	8 (12.5%)	26 (28.0%)	16 (42.1%)	24 (32.0%)
CKD5	42 (13.5%)	1 (2.4%)	2 (3.1%)	15 (16.1%)	5 (13.2%)	19 (25.3%)

anti-PLA2R, anti–phospholipase A2 receptor; CKD5, chronic kidney disease stage 5; DSC, doubling of serum creatinine; IPR, partial remission with immunosuppression; IS, immunosuppression; progression, composite outcome of doubling of serum creatinine, CKD5 or death (whichever is earliest); PR, partial remission; SPR, spontaneous partial remission.

Low: uPCR < 350 mg/mmol and eGFR > 60 ml/min per 1.73 m^2^; Moderate-1: 350 mg/mmol ≤ uPCR < 600 mg/mmol and eGFR > 60 ml/min per 1.73 m^2^; Moderate-2: uPCR ≥ 600 mg/mmol and eGFR ≥ 60 ml/min per 1.73 m^2^; High-1: 300 mg/mmol ≤ uPCR < 600 mg/mmol and eGFR < 60 ml/min per 1.73 m^2^; High-2: uPCR ≥ 600 mg/mmol and eGFR < 60 ml/min per 1.73 m^2^.

#### Change in eGFR During “Watchful Waiting”?

**Figure 2:**
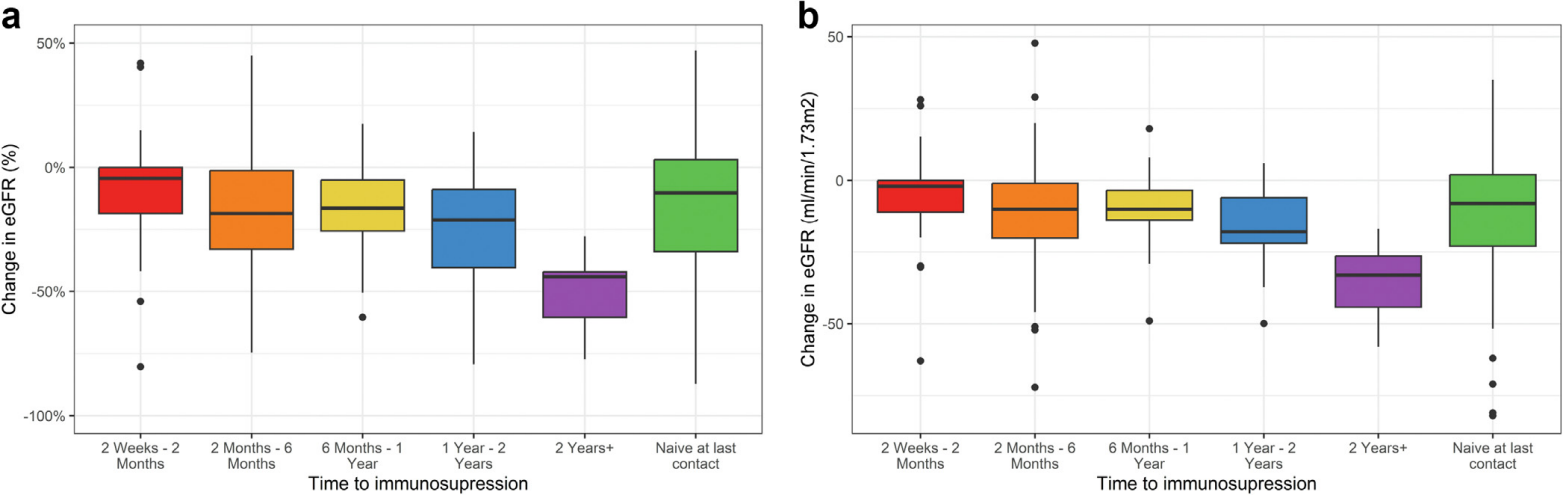
Change in eGFR between baseline and the start of immunosuppression by the length of time to start IS. (a) percentage change, (b) rate of change. eGFR, estimated glomerular filtration rate; IS, immunosuppression.

**Figure 3:**
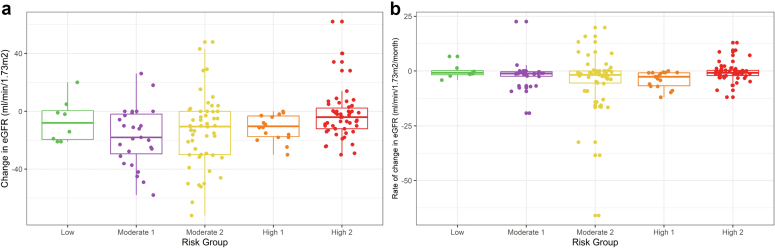
Change in eGFR from the time of biopsy to the initiation of immunosuppression by group (for patients who received immunosuppression). Change in eGFR was significant [*P* = 0.014] and even after accounting for time to IS [*P* = 0.032], with patients in the moderate groups losing the most eGFR. eGFR, estimated glomerular filtration rate; IS, immunosuppression.

In Figure 2, we show the change in eGFR between baseline and the start of immunosuppression based on the length of time to start immunosuppression (Figure 2a: percentage change; and Figure 2b: absolute change). Across the cohort, patients lost an average of 20% of kidney function (eGFR) by the time immunosuppression was initiated. Even in those patients who received immunosuppression within a 6-month period from diagnosis, there was decline in eGFR. Overall, patients who waited the longest until starting immunosuppression treatment lost the highest proportion of their kidney function (∼40% at 2 years or more) and those who waited over 6 months had a median decline in eGFR of 11 ml/min per 1.73 m^2^/yr. Those with much longer times to start immunosuppression treatments continued to lose function in what can be interpreted as a stable rate of decline. A significant association was observed between waiting time to immunosuppression and decline in eGFR (*P* < 0.001) in patients who received immunosuppression. When change was regressed against time, eGFR loss was equivalent to 7.7 ml/min per 1.73 m^2^/yr of watchful wait. The absolute change in eGFR from biopsy to initiation of immunosuppression was higher in the moderate groups, and this change was significant even after accounting for time to immunosuppression (*P* = 0.032) (Figure 3). For those who went on to have immunosuppression, the median eGFR at the time of diagnosis was 64.4 ml/min per 1.73 m^2^ (interquartile range: 40.2–79.8). At the time of immunosuppression, it was 49 ml/min per 1.73 m^2^ (interquartile range: 32.1–71) and at last follow-up it was 52.4 ml/min per 1.73 m^2^ (interquartile range: 36–71.8). The median change in eGFR from diagnosis to immunosuppression was −10 ml/min per 1.73 m^2^ (−20 to −0.03) and from the time of immunosuppression to last follow-up was −6.6 ml/min per 1.73 m^2^ (interquartile range: −18.00 to 0). There was no significant correlation observed between the time to immunosuppression and the rate of eGFR change, and the eGFR at the time of immunosuppression is substantially better than eGFR at the time of biopsy (C = 0.717 vs. C = 0.621) at predicting post immunosuppression outcome.

#### Likelihood of SPR

A total of 118 patients (37.8%) achieved SPR across the cohort. This proportion reduced gradually as per the risk groups; 71.4% in low-risk group to 20% in the high-2 risk group.

**Figure 4:**
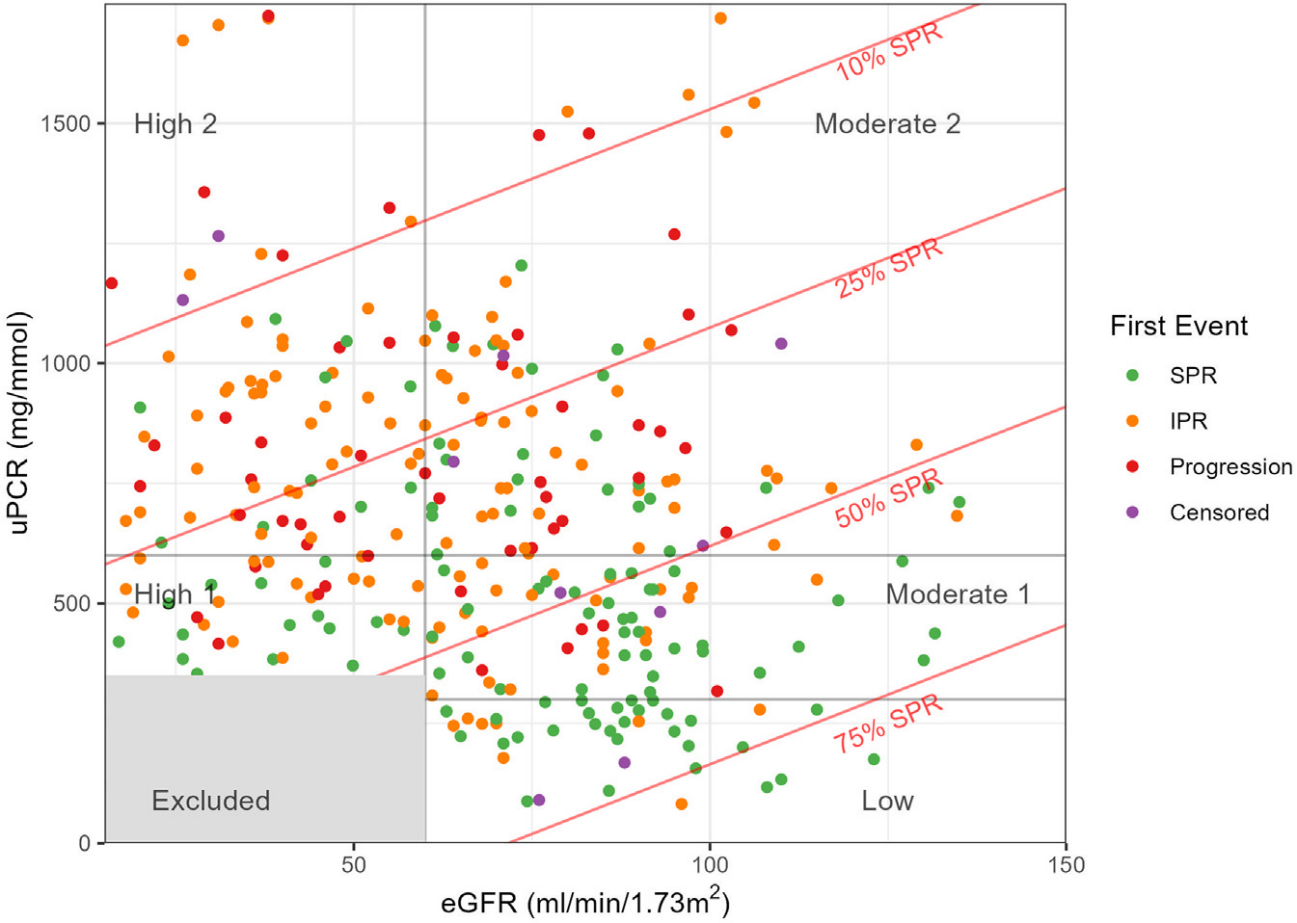
Scatter plot of the eGFR (on x-axis) and uPCR (on y-axis) at baseline. Each point represents the first key outcome per patient, that is, whether it was SPR, IPR, or progression of disease. Areas of the plot that correspond to the risk groups are annotated. The red lines denote the chance of SPR, that is, the set of values of uPCR and eGFR, which in the logistic regression performed, would lead to a predicted chance of SPR of 10%, 25%, 50%, or 75%. Low: uPCR < 350 mg/mmol and eGFR > 60 ml/min per 1.73 m^2^; Moderate-1: 350 mg/mmol ≤ uPCR < 600 mg/mmol and eGFR > 60 ml/min per 1.73 m^2^; Moderate-2: uPCR ≥ 600 mg/mmol and eGFR ≥ 60 ml/min per 1.73 m^2^; High-1: 300 mg/mmol ≤ uPCR < 600 mg/mmol and eGFR < 60 ml/min per 1.73 m^2^; High-2: uPCR ≥ 600 mg/mmol and eGFR < 60 ml/min per 1.73 m^2^. CKD5, chronic kidney disease stage 5; eGFR, estimated glomerular filtration rate; IPR, partial remission with immunosuppression; IS, immunosuppression; PR, partial remission; progression, composite outcome of doubling of serum creatinine, CKD5, or death (whichever is earliest); SPR: Spontaneous partial remission; uPCR, urinary protein-to-creatinine ratio.

In Figure 4, we present a scatter plot visualizing the baseline eGFR and uPCR against the first significant event in the patient journey. The data set includes several outcomes, color-coded for ease of interpretation. The lines plotted across the graph represent the probabilities of SPR as determined by logistic regression analysis. Each line corresponds to a predicted probability for SPR: 10%, 25%, 50%, and 75% (Supplementary Equation 1). These thresholds delineate the likelihood of patients reaching SPR at varying levels of baseline eGFR and uPCR. The progression of these lines demonstrates the inverse relationship between the probability of SPR and higher levels of uPCR, as well as the complex relationship with eGFR. As expected, there is a consistent relationship between both high eGFR and low uPCR in terms of probability of achieving SPR. In Figure 1, Figure 4, we show that patients in the low and moderate-1 risk groups are more likely to go into SPR and conversely; patients in the high-1 and high-2 groups are less likely to achieve SPR. In Table 2, we outline the proportion of patients achieving SPR in the 5 risk groups.

**Figure 5:**
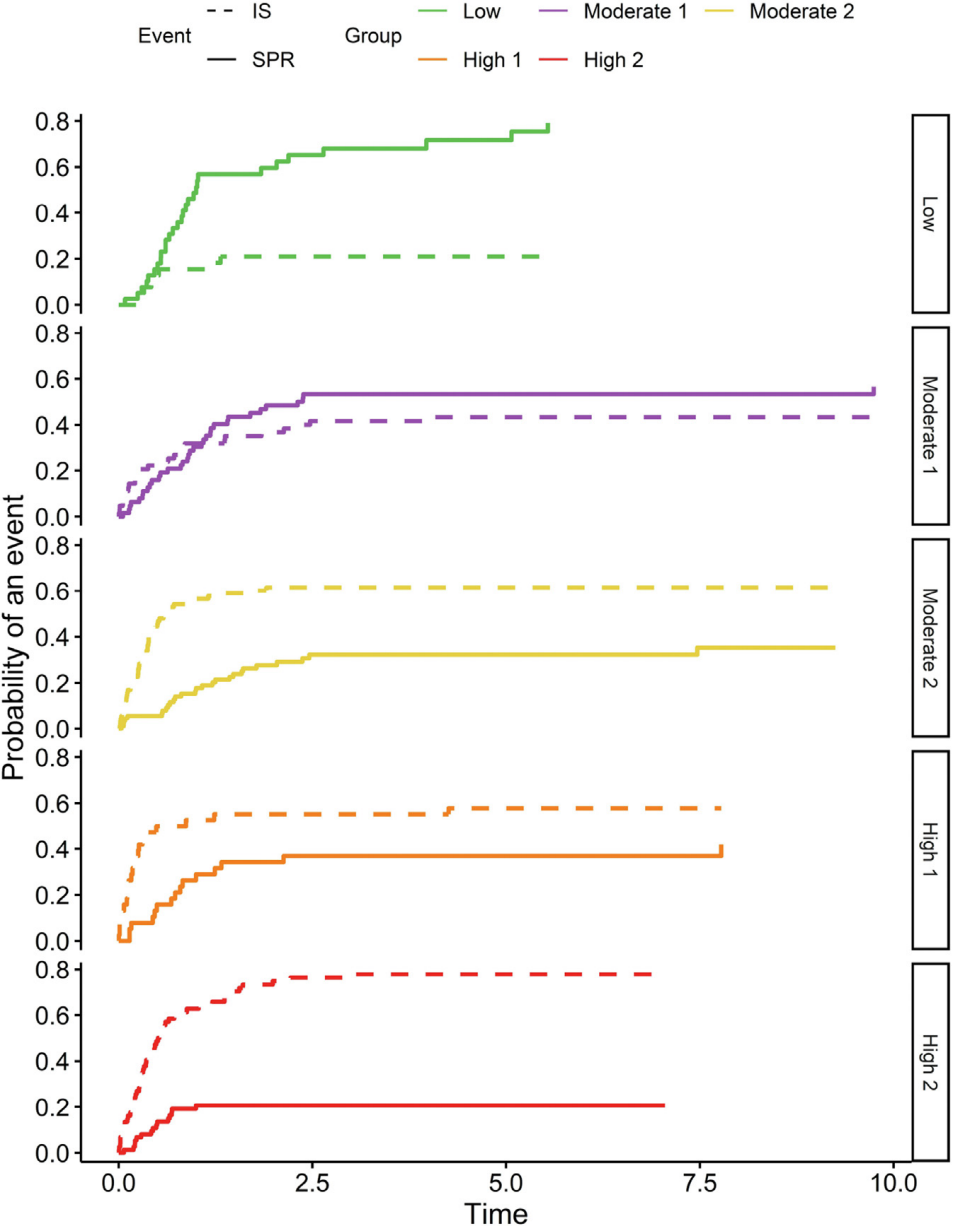
Cumulative incident rate for either spontaneous remission or the need for immunosuppression over time, stratified by risk group.

In Figure 5, we show the cumulative incident rate for either spontaneous remission or the need for immunosuppression by risk group. In the low and moderate-1 risk groups, the rate of SPR is higher than the need for immunosuppression; however, in the higher groups, the need for immunosuppression often comes sooner than SPR.

#### Progression to CKD5

During the median follow-up of 5 years, 78 patients (25%) and 42 patients (13.5%) progressed to doubling of serum creatinine and CKD5, respectively in the whole cohort (Table 3). Rates of doubling of serum creatinine and CKD5 increased gradually as risk category increased. Under a multivariable analysis, the strongest predictor of progression to CKD5 after the start of immunosuppression is eGFR at the start of immunosuppression and there was a minor, but significant benefit to adding in uPCR at immunosuppression. Of the 159 patients who started immunosuppression with eGFR > 15 ml/min per 1.73 m^2^, 22 (13.8%) progressed to CKD5. Decline in eGFR of 10 ml/min per 1.73 m^2^ was associated with 38% increased risk of CKD5 (*P* < 0.001). This model had a concordance of C = 0.717 (0.617– 0.817). By adding uPCR to the model, a concordance of C = 0.739 (0.637–0.841) was noted. Adding further terms (baseline eGFR and uPCR and time to immunosuppression) within the model, led to only marginal and nonsignificant improvement on analysis of variance (C = 0.766, *P* = 0.605). Supplementary Figure S1 shows those in the low and moderate-1 risk groups are more likely to be progression free, with patients in the high-1 and high-2 groups more likley to progress.

## Discussion

“Watchful waiting” in the management of aMN stems from the observation that a subset of patients may achieve spontaneous remission without intervention. The decision to initiate immunosuppressive therapy is therefore influenced by an individualized risk assessment. Although this avoids the risks associated with early immunosuppressive therapy, it could carry the chance of missing an optimal therapeutic window for some patients, particularly in those with severe disease, potentially leading to irreversible kidney damage.^14^ The phenomenon of epitope spreading implies that patients who have had a longer disease course and consequently, a more matured antibody response to the antigen, have worse outcomes.^15^ Our study provides insights, quantifying changes in kidney excretory function during this “watchful wait” period along with the chance of achieving spontaneous remission (and therefore the likelihood of avoiding immunosuppressive treatment) in a large cohort of 312 patients. In 186 patients receiving treatment with immunosuppression, on regression analysis, there was a total loss of 7.7 ml/min per 1.73 m^2^/yr in renal excretory function from diagnosis to the start of immunosuppression. This loss of eGFR was associated with time from biopsy to immunosuppression, and the decline was greatest in those “waiting” beyond 6 months from presentation.

Previous studies have documented the progression of kidney disease in untreated aMN, with a significant number of patients progressing to severe renal failure (creatinine > 400 μmol/l or end-stage kidney disease) within 2.5 years of diagnosis.^4^^,^^6^ The recent prospective, real-world study investigating the use of rituximab showed a significant reduction in the annual rate of eGFR decline once treatment started, from 13.9 to 1.7 ml/min per 1.73 m^2^/yr.^16^ Our study corroborates these findings as a whole, adding further detail on renal function changes at the cohort level and across different risk groups. Importantly, we highlight the strongest predictor of renal outcome as the eGFR at the start of treatment, regardless of the previous trajectory or time to the start of immunosuppression. This, in addition to the potential for achieving SPR, provides clinicians and patients with a better estimation of the trade-off with or without intervention using immunosuppression. Patients with an eGFR < 60 ml/min per 1.73 m^2^ (those in the high-1 and high-2 risk groups), which constituted 36% of our cohort, face the most significant risk. These groups had a median eGFR of 39 ml/min per 1.73 m^2^ at presentation, and their potential for decline without achieving spontaneous remission carries substantial medium- to long-term implications.

The heterogeneity in aMN's clinical presentation, with many patients initially presenting with preserved renal function, complicates the decision to initiate treatment. Analysis of changes in renal function in patients with aMN reveals a complex picture. Previous studies have shown higher rates of spontaneous remission in those with lower baseline proteinuria and ostensibly normal renal function; however, this is not universal and some patients will still achieve spontaneous remission despite significant proteinuria, and some will still reach end-stage kidney disease even with immunosuppression.^3^^,^^14^ Here, rates of SPR and decline in renal function varied based on grouping: 71% of SPR and only 2.4% of CKD5 the low-risk group, compared with 20% achieving SPR and 25% developing CKD5 in the high-2 risk group during the follow-up. Kidney function can decline during the period before treatment begins; however, starting treatment early could potentially slow this decline if outcome at last follow-up is dependent on the eGFR at the start of treatment. It is plausible that starting treatment earlier could potentially offer benefits despite the risks. However, this does need further evaluation in prospective studies to confirm. Predicting prognosis at presentation would be crucial in determining initiation of disease-modifying treatment with immunosuppressive treatment. Despite several models being proposed over the past 20 to 30 years to help predict prognosis, progression of CKD remains a possibility with incidence of ESKD being a reality in a significant proportion of patients.^17^, ^18^, ^19^, ^20^, ^21^, ^22^, ^23^ Emerging research is continually refining our understanding of aMN's natural history and the impact of various treatment strategies. For example, recent studies are exploring the role of less toxic agents such as rituximab in patients with progressive or advanced kidney dysfunction and the potential for personalized medicine approaches based on specific biomarkers.^24^ In addition, the psychological impact of “watchful wait” on patients, who may feel anxious about their untreated disease, should not be underestimated, particularly given the effect ongoing nephrotic syndrome can have on their quality of life .^25^

Our study has all the inherent limitations of a retrospective study. However, the rare nature of MN has required a pragmatic approach to many studies; using a multicenter study attempted to mitigate some of these issues and allowed for a greater cohort and generalizability. The pragmatic nature of the study has led to some subgroups being naturally imbalanced and events being underpowered with resultant statistical nonsignificance, despite seemingly considerable hazard ratios. Evidence for baseline anti-PLA2R in determining prognosis is conflicting. We have shown in our earlier study of the same cohort, that it does not add further value beyond conventional markers in the form of uPCR and eGFR. Recently, studies evaluating the role of baseline anti-PLA2R in predicting prognosis showed conflicting results as well.^9^^,^^17^^,^^26^ We argue that the tight association of anti-PLA2R with clinical parameters such as proteinuria and eGFR mean that they lose additional value in influencing prognosis when adjusted for baseline renal markers. Evaluating the role of serial anti-PLA2R antibody titers was beyond the scope of this study and large scale studies are desperately needed to refine their utility. This study was not designed to assess efficacy of individual treatment options or regimens and therefore, we did not analyze the effect of treatment on the outcomes, particularly because patients may have received multiple regimens during the course of their disease. Taking a pragmatic approach, immunosuppression was not separated and was considered globally. Competing risk analysis was explored or attempted but not appropriate given the numbers of patients with events. Data were not collected for interim appointments and only at key milestones. In a future study, we will aim to collect these data from electronic patient record to collate a richer data set. Another limitation is that it is an assumption that the worsening of eGFR is purely due to the immunological disease state or as a consequence of chronic scarring or severe hypoalbuminemia, including renal vein thrombosis, infections, etc., as opposed to other unrelated phenomena.

The management of aMN remains a challenging and dynamic field. Although significant strides have been made in understanding and treating the condition, key questions remain, particularly regarding the optimal timing for initiating treatment. Here, we show that far from being a benign monitoring period, there is a consequence to the “watchful wait” approach, with a continued renal decline prior to the start of treatment in a significant proportion of patients with aMN. We show that those patients in the high-risk categories have the most to benefit from earlier treatment given the lesser likelihood of achieving spontaneous remission; with the data revealing that the longer it takes to start treatment, the worse the renal decline can be; and that the final outcome is significantly correlated to eGFR at treatment. It is likely that newer less toxic treatments such as rituximab could make the case for earlier treatment with a better risk benefit profile. In addition, by risk stratifying based on the categories we have presented here, a more personalized approach can be taken to avoid future harm. This study brings to the fore the need for a more refined risk stratification tool and pragmatic and patient-centered trial designs with a focus on exploring personalized treatment approaches.

## Disclosure

All the authors declared no competing interests.

## Data Availability Statement

Data restrictions have been imposed by Manchester Foundation Trust Research and Innovation department. Data can be requested through our research and innovation department in Manchester Foundation trust (R&D.Applications@mft.nhs.uk).

## Supplementary Material

Supplementary File (PDF) **Supplementary Equation 1.** Equation used for logistic regression analysis for the predicted probability for SPR at 10%, 25%, 50%, and 75% – see Figure 4. **Figure S1.** Kaplan-Meier plot showing progression free probability based on risk categories.

## Footnotes

Supplementary File (PDF)
**Supplementary Equation 1.** Equation used for logistic regression analysis for the predicted probability for SPR at 10%, 25%, 50%, and 75% – see Figure 4. **Figure S1.** Kaplan-Meier plot showing progression free probability based on risk categories.
