# Supplementary material for: Impact of Time-To-Treatment on Outcomes in Autoimmune Membranous Nephropathy
Source: Kidney Int Rep. 2025 Apr 9;10(6):1907–16. doi: 10.1016/j.ekir.2025.04.005 (PMC12231015; doi:10.1016/j.ekir.2025.04.005)
Supplement: Supplementary File (PDF) — Supplementary Equation 1. Equation used for logistic regression analysis for the predicted probability for SPR at 10%, 25%, 50%, and 75% – see Figure 4. Figure S1. Kaplan-Meier plot showing progression free probability based on risk categories. [file mmc1.pdf]

Supplementary equation 1

$$log(OR) = 0.094 + 0.014eGFR - 0.00241uPCR$$

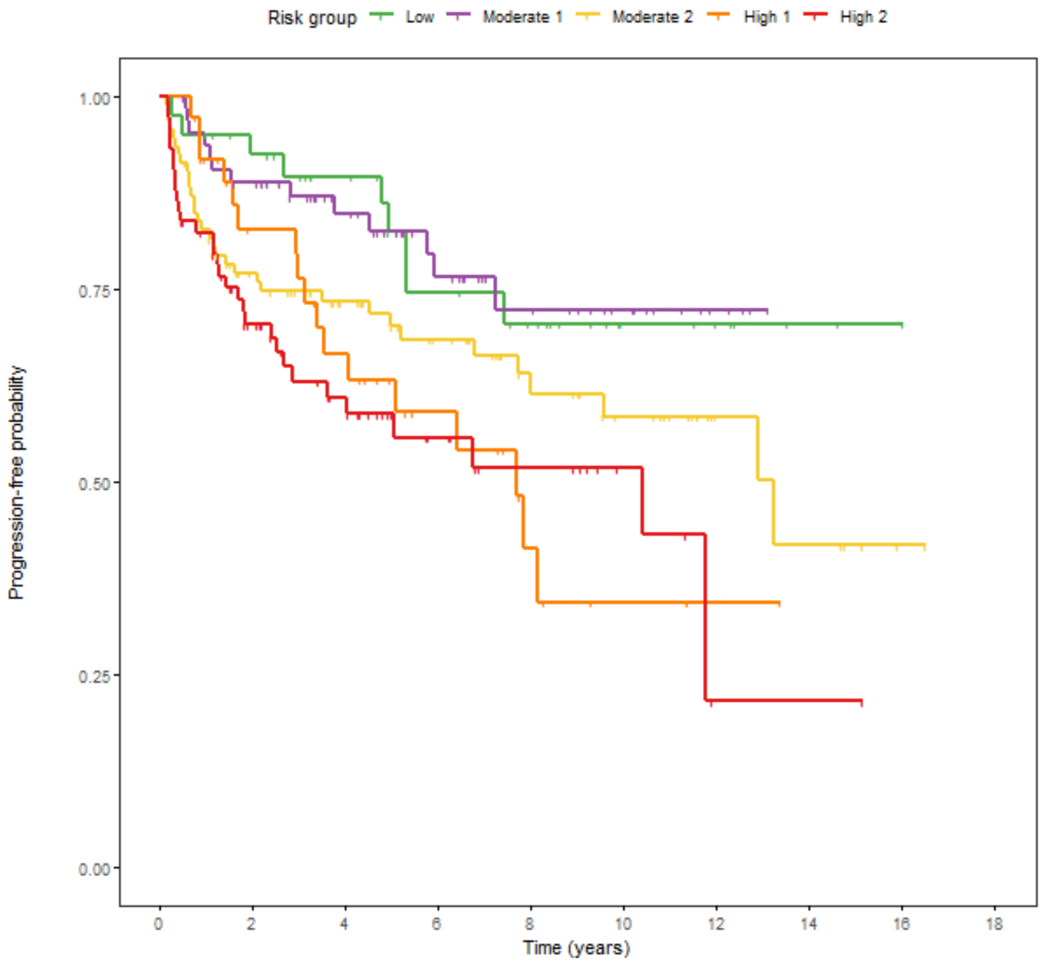

Number at risk

Risk group	0	2	4	6	8	10	12	14	16	18
Low	42	35	27	19	15	7	6	2	1	0
Moderate 1	64	55	38	26	17	11	4	0	0	0
Moderate 2	93	63	50	39	24	18	7	5	1	0
High 1	38	26	20	12	6	3	1	0	0	0
High 2	74	43	28	16	11	6	1	1	0	0

Time (years)

Supplementary figure 1 – Kaplan-meier plot showing progression free probability based on risk categories.
